# Mini-review: Harm reduction strategies among people who intentionally use fentanyl

**DOI:** 10.1016/j.abrep.2025.100615

**Published:** 2025-05-24

**Authors:** Jacob Paredes, Ashish Sandhu, Shutong Huo, David S. Timberlake

**Affiliations:** Joe C. Wen School of Population and Public Health, University of California, Irvine, 856 Medical Sciences, Irvine, CA 92697, USA

**Keywords:** Fentanyl, Harm Reduction, Intentional Use

## Abstract

**Background:**

Most published studies aimed at curtailing the morbidity and mortality associated with the current U.S. opioid epidemic have focused on unintentional exposure to fentanyl through heroin, cocaine, and other substances. Yet, there is mounting evidence that the intentional use of fentanyl is a key contributor to the current epidemic. In this mini-review we will discuss the harm reduction methods employed by people who intentionally use fentanyl (PWIUF) from a handful of articles found through PubMed that included PWIUF that were published between January 2013 to January 2024.

**Results:**

The perceived harm reduction strategies included co-use of stimulants, dose reduction, buying from a trusted dealer, drug checking, conducting a test shot, carrying naloxone, using fentanyl with others, and switching from injection to another route of administration (e.g., smoking). Some of these harm reduction strategies are frequently used by persons who inject heroin but future research on the efficacy of these harm reduction strategies for fentanyl use is warranted. It is important to note that co-use of stimulants has been associated with an increase in overdose.

**Conclusion:**

Future research is needed to understand intentional fentanyl use, perceived harm reduction strategies and the efficacy of these strategies with the advent of stronger opioids. Future research about PWIUF should consider novel study designs such as the ecological momentary assessment for more granular observation of the use of harm reduction strategies.

## Highlights

• People who intentionally use fentanyl (PWIUF) are an understudied population.
• The population of PWIUF in North America has increased.
• PWIUF use several perceived harm reduction strategies to reduce the risk of overdose.

## 1 Introduction

Media coverage of the current U.S. opioid epidemic has largely focused on the deaths of youths and young adults who inadvertently consumed fentanyl through counterfeit pills and other substances (Ibarra et al., 2023). Yet only 5 % of overdoses in 2021 have been linked to counterfeit pills, suggesting other sources of non-prescription fentanyl are driving the epidemic (O’Donnell, 2023). Similarly, current harm reduction literature mainly focuses on the avoidance of fentanyl and unintentional fentanyl use but does not address people who intentionally use fentanyl (Wakeman, 2019). We define “intentional use of fentanyl” as the deliberate use of fentanyl or fentanyl-adulterated drug with the understanding or expectation that the drug contains fentanyl or fentanyl analog. Intentional use of fentanyl has been documented in the substance use literature but is less visible to the public due to homelessness or incarceration of the population (Duhart Clarke et al., 2022, Foglia et al., 2021, Silverstein et al., 2019, Urmanche et al., 2022). Additional elements may be increasing the rate of intentional use of fentanyl, such as the saturation of fentanyl in local drug markets (Mars et al., 2019), the ensuing need for strong potency to overcome opioid tolerance (McLean et al., 2019), and the cheaper price of fentanyl (Urmanche et al., 2022). Some users prefer fentanyl over other opioids but acknowledge the dangers of overdosing (Silverstein et al., 2019). Thus, researching and promoting effective harm reduction practices in this vulnerable and overlooked population of people who intentionally use fentanyl (PWIUF) is a health equity issue that needs our urgent attention (Kapadia, 2023). Harm reduction is a public health alternative that seeks to reduce the harms of drug use in a manner that is more accessible than complete cessation (Marlatt, 1996). Perceived harm reduction strategies are strategies that PWIUF may employ with the expectation that these strategies will protect them from overdose. This mini-review aims to review the literature on perceived harm reduction practices used by PWIUF. We will discuss the harm reduction methods employed by PWIUF from 15 articles found through PubMed that reported on PWIUF and were published between January 2013 to January 2024.

## 2 People who intentionally use fentanyl

The topic of intentional fentanyl use is of growing concern. The percentage of U.S. residents who misused prescription or illicit fentanyl was 0.4% in 2022 according to the National Survey on Drug Use and Health (SAMHSA, 2023). This statistic from SAMHSA does not distinguish intentional versus unintentional fentanyl use (SAMHSA, 2023). To better understand rates of intentional fentanyl use in North America, we may look at the findings of individual geographically limited studies. A study conducted from 2021 to 2022 found that among a sample of people who inject drugs in New York city, 18 % of participants reported intentional use (McKnight et al., 2023). A separate study from San Francisco observed that illicit fentanyl had replaced heroin (Kral et al., 2021). And another study conducted in the SouthEastern United States also provides a similar observation (Peiper et al., 2019). Furthermore, A Canadian found that preference for fentanyl is growing among people who inject opioids from 4.4 % to 6.6 % across a single year (Ickowicz et al., 2022).

Intentional fentanyl use is associated with white race/ethnicity, male, homelessness, unemployment and incarceration, younger age, poly drug use, and living within counties with high fentanyl sales (Foglia et al., 2021, Tsang et al., 2024). A preference for the effects of fentanyl, including its rapid onset of euphoria and relief from withdrawal symptoms, increases the likelihood of intentional fentanyl use (Comer and Cahill, 2019, Gryczynski et al., 2019, Macmadu et al., 2017). Additionally, individuals in areas with high fentanyl exposure require stronger drugs due to opioid tolerance (Cicero et al., 2018, Macmadu et al., 2017, Mars et al., 2018). This preference is particularly prevalent among white populations, possibly due to their higher likelihood of receiving mental illness diagnoses and a lower tendency to acknowledge the severe withdrawal effects of fentanyl, which are associated with risky drug use (Foglia et al., 2021, Sehgal, 2012).

One important factor of the growth of fentanyl in the illicit market is cost, in that fentanyl is 10 times cheaper than heroin at wholesale (Rothberg & Stith, 2018). Since fentanyl has a lower cost than heroin, switching to fentanyl from another opioid is a “financially conscientious decision” (Urmanche et al., 2022). Another factor in the growth of fentanyl is the abundant adulteration of illicit drugs with fentanyl (Silverstein et al., 2019). Unintended use of fentanyl such as fentanyl adulterated heroin can increase tolerance to opioids, shifting people who use other opioids/opiates to synthetic opioids (McKnight et al., 2023). For instance, a participant in a study conducted by Silverstein et al. stated, “Whether I realized it or not I was doing it (fentanyl) when it first came out so then my body got used to that” (Silverstein et al., 2019).

## 3 Perceived harm reduction strategies

### 3.1 Co-using stimulants

Co-use of stimulants with opioids is not new as we have observed cocaine co-used with heroin (Daniulaityte et al., 2022). In the age of fentanyl, cocaine continues to be co-used with fentanyl but namely of interest is in the combination of methamphetamines and fentanyl known among some as a “goofball” (Daniulaityte et al., 2022, Duhart Clarke et al., 2022, Lukac et al., 2022, Mazhnaya et al., 2020). PWIUF may co-use with methamphetamines to counteract the strong drowsy effect of fentanyl (Duhart Clarke et al., 2022). Another reason is also the belief that the stimulant will counteract an opioid overdose that would be induced by fentanyl, with one interviewee's rationale of using methamphetamine before using heroin/ fentanyl to keep her heart rate up so she does not overdose (Daniulaityte et al., 2022, Duhart Clarke et al., 2022, Lukac et al., 2022). Of note, this co-use of fentanyl and stimulants has been linked to an increase in overdose deaths, therefore this perceived harm reduction strategy may not reduce harm but rather may increase the risk of harm (Hoopsick and Andrew Yockey, 2023, Rawson et al., 2023).

### 3.2 Reducing dosage

The reduction of dosage entails reducing the actual dosage or using the drug more slowly (Karamouzian et al., 2018, Krieger et al., 2018, Park et al., 2020, Tobias et al., 2024). Given that fentanyl is 50x stronger than heroin (Ibarra et al., 2023), using less fentanyl is a logical strategy. Though with illicit fentanyl, potency can vary considerably between batches as a likely function of fentanyl analogs such as Carfentanil, which is 100x more potent than fentanyl (Zawilska et al., 2021). Due to variability of potency, simply using less may not be enough to avoid an overdose. Still the intention to reduce dosage is associated with lower odds of overdose (Karamouzian et al., 2018), therefore reducing dosage may reduce harm.

### 3.3 Having a trusted dealer

Purchasing opioids from a trusted dealer is another strategy observed among PWIUF and heroin users (Carroll et al., 2020, Duhart Clarke et al., 2022, LaForge et al., 2022). For the latter group, the strategy is typically used to avoid fentanyl exposure (Carroll et al., 2017). The dealer plays a role in providing essential information that PWIUF need to know, such as fentanyl content and fentanyl dosage so the consumer can make informed decisions about their use (Carroll et al., 2020, Duhart Clarke et al., 2022). The dealer may even play a larger role in preventing death from overdose as reported in the study by Carroll et al. (Carroll et al., 2020). Having a trusted dealer may reduce harm from intentional fentanyl use. The authors of this study observed a relationship between trusted dealers and reduced risk of substance use related harm (Carroll et al., 2020).

### 3.4 Drug checking

Another strategy used by PWIUF is to check the drug using fentanyl test strips or visiting a drug checking site to identify the presence of fentanyl within their drugs (Bailey et al., 2023, Goldman et al., 2019, Karamouzian et al., 2018, Krieger et al., 2018, LaForge et al., 2022, Park et al., 2020, Tilhou et al., 2022, Tobias et al., 2024). PWIUF are more equipped to make informed decisions about the dosage of their drug use by knowing the content of their drugs. Fentanyl test strips can provide important information about the content of the drug but it is limited in that the fentanyl test strip does not provide information about the amount nor potency of fentanyl (LaForge et al., 2022). Drug checking may reduce harm from intentional fentanyl use. While immediate knowledge of amount and potency would be more ideal for PWIUF, it is shown that fentanyl test strip use is associated with other harm reduction strategies such as injecting slowly, conducting a test shot, and carrying naloxone (Goldman et al., 2019, Krieger et al., 2018).

### 3.5 Conducting a test shot

Knowing the amount and potency of fentanyl is vital information for PWIUF. Conducting a test shot is a strategy seen among PWIUF, which is to use a much smaller amount of the drug to feel and better understand the strength of the drug before using more of the drug (Carroll et al., 2017, Duhart Clarke et al., 2022, Goldman et al., 2019, Mars et al., 2018, McKnight et al., 2023, Park et al., 2020). By initially conducting a test shot, one may be able to make a better informed decision on how much of the drug to continue to use (Duhart Clarke et al., 2022, Mars et al., 2018). None of the reviewed studies assessed efficacy of test shots, which raises the question of whether test shots will still be a viable harm reduction strategy as stronger synthetic opioids come onto the market.

### 3.6 Carrying naloxone

Another strategy is to carry naloxone (French et al., 2023, LaForge et al., 2022, McKnight and Des Jarlais, 2018), an opioid receptor blocker that is a safe and effective method of reversing opioid overdoses. Scholars, however, find conflicting results regarding the relation between Naloxone Access Laws (NALs) and opioid overdose mortality. Two studies concluded that states with NALs experienced a substantial decline in opioid overdose deaths before 2014 (McClellan et al., 2018, Rees et al., 2019). However, as the study period extended to 2015 or 2016 (coinciding with the latest rise in fentanyl deaths), three studies found no difference in overdose fatalities between states with and without NALs (Abouk et al., 2019, Atkins et al., 2019, Doleac and Mukherjee, 2018). It has been observed that multiple doses of naloxone may be required to reverse a fentanyl overdose (Abdelal et al., 2022, Fairbairn et al., 2017, Mars et al., 2018). However, concentrated naloxone doses that produce high bioavailability cost more and impede their accessibility to people who use drugs (Kim et al., 2019). Another factor to consider is that stigma about drug use may dissuade people who are at risk of witnessing an overdose from carrying naloxone (Spadaro et al., 2023). Overall, policies like take-home naloxone programs, which provide naloxone to people who use opioids and their families, have been shown to reduce mortality rates (Kim et al., 2019, McDonald et al., 2017).

### 3.7 Using with others

Relatedly, PWIUF may use drugs with others. By using drugs with others, naloxone and further aid may be more readily administered in the event of an overdose. People may use among friends/ family, visit a safe injection site (safe consumption site/ drug consumption rooms) or at least ask someone to check on them at a later time (Bouvier et al., 2017, Duhart Clarke et al., 2022, French et al., 2023, Kennedy et al., 2020, O’Rourke et al., 2019, Park et al., 2020, Ritchie and Ghosh, 2022, Tobias et al., 2024). Experiences from OnPoint, the United State’s first and only government supported overdose prevention site, shows that using around others does provide a safety net in the event of an overdose (McAteer et al., 2024).

### 3.8 Switching from injection

Another strategy is to change routes for administering fentanyl from injecting to either nasal insufflation (i.e. snorting), or smoking (Duhart Clarke et al., 2022, Kral et al., 2021, LaForge et al., 2022). The rationale for switching from injection to another administration route is the perception that other routes are safer (Duhart Clarke et al., 2022, LaForge et al., 2022). For instance, one participant in a study conducted by LaForge et al. (2022) stated that by smoking the drug, one might fall asleep before passing out while with injection use, one might simply pass out. Comparatively, a participant in a study conducted by (Duhart Clarke et al., 2022), shared their experience that insufflation of fentanyl does not reduce risks of overdose when using fentanyl. Further research is needed to understand this perceived harm reduction strategy.

## 4 Conclusion

With the danger fentanyl poses, people who intentionally use fentanyl may employ several perceived harm reduction strategies to reduce their risk of death from opioid overdose. Strategies taken may include co-use of stimulants, buying from a trusted dealer, reducing dosage, conducting a test shot, drug checking, carrying naloxone, using with others, and/ or changing administration routes. Many of the strategies discussed are not new to the opioid landscape such as using less or slower, conducting test shots, drug checking, carrying naloxone, using around others, and changing administration routes (Curtis and Guterman, 2009, Coalition, 2024). Some perceived harm reduction strategies, namely the co-use of stimulants, has been related to an increase in overdose deaths. More empirical studies about fentanyl use, perceived harm reduction strategies, and efficacy of these perceived harm reduction strategies among people who intentionally use fentanyl is warranted as new stronger drugs come onto the market. As more studies are published about intentional fentanyl use, a more complete scoping review should be conducted. Future research may also seek to study how harm reduction strategies implemented by governments may compare to harm reduction strategies conducted by PWIUF. As previously stated, it is unclear how many people are intentionally using fentanyl, therefore a quantitative study about the current prevalence of intentional fentanyl use is also warranted.

As shown in Table 1, a majority of the studies, 12 out of 15, were either of cross-sectional or semi-structured design. The study of intentional fentanyl use would benefit from ecological momentary assessment study methodology. Ecological momentary assessment uses technology, such as smartphones, to collect real-time quantitative and/or qualitative data in mostly regular intervals over time to better understand behavior in real-life environments (Shiffman et al., 2008). Ecological momentary assessment studies would benefit the study of fentanyl use by providing granular information about the context and individual differences about harm reduction strategy use and drug use, such as the social/ physical environment, mood, and pain that precede drug use (Lorvick et al., 2023).

**Table 1:** Intentional fentanyl use harm reduction strategies initial reviewed articles.

Lead author, Year	Study design	Recruitment	Sample size	Harm reduction strategy
Bailey et al., 2023	Longitudinal cohort	• Street Outreach	426	• Drug Testing
Bouvier et al., 2017	Cross-sectional	• Internet Recruitment • Physical Advertisements• Word of Mouth	54	• Use with Others
Carroll et al., 2017	Mixed-methods: cross-sectional/semi-structured interview	• Community-Based Health Clinics • Harm-Reduction Outreach • Emergency Departments• Needle Exchange Programs	149 Surveys47 Interviews	• Test Shot
Duhart Clarke et al., 2022	Qualitative, semi-structured	• Needle Exchange Programs	28	• Trusted Dealer • Co-use Stimulants • Use with Others • Switch from Injection • Test Shot
French et al., 2023	Qualitative, semi-structured	• Hospital	21	• Carry Naloxone• Use with Others
Karamouzian et al., 2018	Cross-sectional	• Self-Referral• Street Outreach	1411 drug checks	• Drug Testing • Reduce Dosage
Kennedy et al., 2020	Prospective cohort study	• Needle Exchange Programs • Peer Recovery Support Specialists• Snowball Sampling	319	• Use with Others
LaForge et al., 2022	Qualitative, semi-structured	• Harm Reduction Supply Distribution Site	34	• Drug Testing • Switch from Injection • Naloxone • Trusted Dealer
Lukac et al., 2022	Cross-sectional	• Harm Reduction Outreach Programs	574	• Co-use Stimulants
Mazhnaya et al., 2020	Cross-sectional	• Harm Reduction Outreach Programs• Street Outreach	311	• Co-use Stimulants
McKnight and Des Jarlais, 2018	Cross-sectional	• Needle Exchange Programs	57	• Test Shot • Naloxone
O’Rourke et al., 2019	Cross-sectional	• Harm Reduction Program• Street Outreach	373	• Use with Others
Park et al., 2020	Prospective cohort study	• Street Outreach	103	• Use with Others • Drug Testing • Reduce Dosage • Test Shot
Tilhou et al., 2022	Mixed-methods: cross-sectional/semi-structured interview	• Needle Exchange Programs	370	• Drug Testing
Tobias et al., 2024	Cross-sectional	• Harm Reduction Outreach Programs	537	• Use with others • Drug Testing • Reduce Dosage

PWID. People Who Inject Drugs.

Summary of the 15 initial articles’ study designs, harm reduction strategies expressed, and recruitment strategies.

This mini-review is not free from limitations. This mini-review used only PubMed for article retrieval, which may have omitted relevant literature from databases focused on psychological or social sciences. We recommend that more comprehensive systematic or scoping reviews include databases such as PsycNet and SCOPUS. In addition, we did not discuss the heterogeneity of drug supplies, drug-use cultures, and regulatory environments across North America. We encourage future studies to examine these regional differences to support more tailored and effective harm reduction approaches.

To continue reducing opioid overdose deaths, a more comprehensive understanding about the efficacy of perceived harm reduction strategies among people who intentionally use fentanyl is paramount. Future studies about utilized harm reduction strategies among this population, can inform public health decisions about community harm reduction resources, interventions, and education that will be more appropriate for people who intentionally use fentanyl.

## CRediT authorship contribution statement

**Jacob Paredes:** Writing – review & editing, Writing – original draft, Project administration, Methodology, Investigation, Formal analysis, Data curation, Conceptualization. **Ashish Sandhu:** Writing – review & editing, Investigation, Formal analysis, Data curation. **Shutong Huo:** Writing – review & editing, Writing – original draft, Formal analysis. **David S. Timberlake:** Writing – review & editing, Supervision, Conceptualization.

## Declaration of competing interest

The authors declare that they have no known competing financial interests or personal relationships that could have appeared to influence the work reported in this paper.

## Data Availability

No data was used for the research described in the article.
